# Bioactive Properties of Peptides and Polysaccharides Derived from Peanut Worms: A Review

**DOI:** 10.3390/md20010010

**Published:** 2021-12-22

**Authors:** Yi Qi, Jingyi Zhou, Xiaoqin Shen, Meram Chalamaiah, Simin Lv, Hui Luo, Liang Chen

**Affiliations:** 1 The Marine Biomedical Research Institute, Guangdong Medical University, Zhanjiang 524023, China; qiyi7272@gdmu.edu.cn (Y.Q.); nancy197@163.com (J.Z.); luohui@gdmu.edu.cn (H.L.); 2 Marine Chinese Medicine Branch, National Engineering Research Center for Modernization of Traditional Chinese Medicine, Zhanjiang 524023, China; 3 College of Pharmacy, Guangdong Medical University, Zhanjiang 524023, China; sxq11192021@163.com; 4 4-10 Ag/For Centre, Department of Agricultural, Food and Nutritional Science (AFNS), University of Alberta, Edmonton, AB T6G 2P5, Canada; chalamaiah.m@gmail.com; 5 Guangdong Runyuan Zhongtian Biological Technology Co., Ltd., Dongguan 523808, China; lvsimin88@126.com

**Keywords:** marine worms, sipunculids, bioactive properties, peptides, polysaccharides

## Abstract

Peanut worms (Sipunculids) are unsegmented marine worms that usually inhabit shallow waters. Peanut worms are good source of bioactive compounds including peptides and polysaccharides. Many recent studies have investigated the bioactive properties of peptides and polysaccharides derived from peanut worms in order to enhance their applications in food and pharmaceutical industries. The peptides and polysaccharides isolated from peanut worms have been reported to possess anti-hypertensive, anti-oxidant, immunomodulatory, anti-inflammatory, anti-cancer, anti-hypoxia and wound healing activities through the modulation of various molecular mechanisms. Most researchers used in vitro, cell culture and animal models for the determination of bioactivities of peanut worm derived compounds. However, studies in humans have not been performed considerably. Therefore, it is important to conduct more human studies for better utilization of marine bioactive compounds (peptides and polysaccharides) derived from peanut worms. This review mainly focuses on the bioactive properties of peptides and polysaccharides of peanut worms and their molecular mechanisms.

## 1. Introduction

Peanut worms are a group of unsegmented marine worms that belong to the invertebrate phylum Sipuncula. There are about 162 species of peanut worms. They are mostly under 10 cm long and live in shallow waters. Peanut worms are edible and considered as a delicacy in several southeast Asian countries including China, Philippines and Vietnam [1]. Some peanut worms (e.g., *Sipunculus nudus*) have been used in traditional Chinese medicine for the treatment/management of various ailments including hypertension, neurosis, coughing with dyspnea, nocturia, carbuncles, sternalgia, physical weakness, tuberculosis and regulating the functions of stomach and spleen [1,2].

Nutritional composition of foods plays an important role in providing essential nutrients for maintaining good health. The nutritional/chemical composition of peanut worms has been investigated [1,3]. The major components of peanut worms are proteins, carbohydrates and ash. For example, the protein content of *P. esculenta* was found to be 74.5% with high quality amino acids, and carbohydrates were 6.2% [3]. Since peanut worms contain significant quantities of nutritional and bioactive compounds, it is important to exploit the peanut worms for identification and isolation of active bioactive compounds for various food and pharmaceutical applications.

Many recent investigations have demonstrated the isolation of diverse peptides and polysaccharides, with various biological properties such as anti-oxidant, anti-cancer, immunomodulatory, anti-inflammatory and anti-hypertensive activities, from a number of marine organisms with great potential for industrial and therapeutic applications [4,5,6]. Food derived bioactive peptides are usually short chains of amino acids and generally possess 2–20 amino acid residues. Bioactive peptides are inactive within the sequence of parent protein. However, the bioactive peptides can be generated by using in vitro enzymatic hydrolysis, fermentation and during food processing and gastrointestinal digestion. In vitro enzymatic hydrolysis is the most widely used method for the production of bioactive peptides from various food sources. Bioactive peptides are easily digestible and absorbable with less or no side effects [7]. Therefore, recently bioactive peptides have attracted great interest among scientists and consumers due to their health benefiting properties [8]. Numerous bioactive peptides with diverse health promoting properties have been reported from various marine species and other food sources including fish, molluscs, crustaceans, algae, milk, egg and plant sources [7,9,10].

Polysaccharides are complex natural macromolecular polymers composed of monosaccharide units linked by glycosidic bonds. Polysaccharides are widely present in plants, animals, microorganisms and marine organisms and play a vital role in the development of living organisms [11]. In recent years, polysaccharides derived from natural sources have attracted great attention due to their biocompatibility, non-toxicity, biodegradability and applications in food and pharmaceutical industries [12]. It has been documented that polysaccharides isolated from natural edible sources exhibit significant bioactive properties [13].

**Figure 1:**
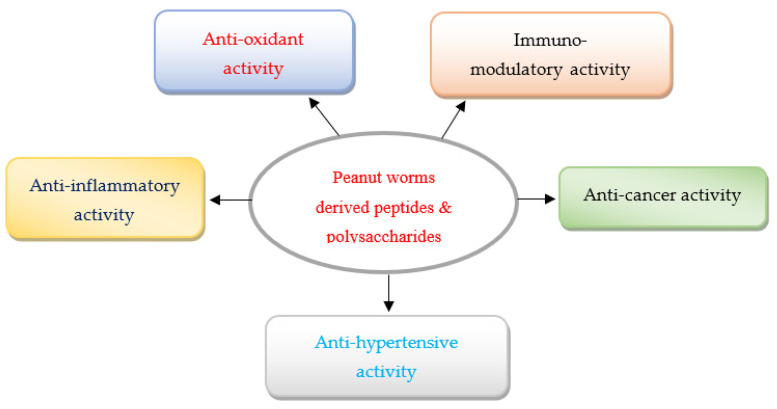
Various major bioactivities of peptides and polysaccharides derived from peanut worms.

Several recent studies have demonstrated that the peptides and polysaccharides obtained from peanut worms possessed numerous health benefiting functions, namely, anti-inflammatory, anti-hypertensive, immunomodulatory, anti-cancer, wound healing, anti-oxidant and anti-hypoxia activities [14,15,16,17,18,19,20] (Figure 1). However, the biological properties of peptides and polysaccharides of peanut worms have not been reviewed in the literature so far. This prompted the authors to review the literature on various bioactivities of peptides and polysaccharides derived from peanut worms and their molecular mechanisms of action.

## 2. Anti-Oxidative Properties of Peptides and Polysaccharides Derived from Peanut Worms

Cellular respiration in humans and other aerobic organisms generates reactive oxygen species (ROS) and free radicals. The anti-oxidant mechanisms (e.g., superoxide dismutase, catalase and glutathione peroxidase) in the body neutralize the excess production of free radicals and thereby maintain the balance between antioxidants and oxidative stress. The ROS and free radicals contain unpaired electrons and cause damage to important cellular components including lipids, proteins and DNA. The over production of ROS and free radicals has been linked to various diseases such as cancer, diabetes, atherosclerosis, Alzheimer disease and cardiovascular disorders [21,22,23]. Recently, there has been great interest in identification and isolation of anti-oxidant bioactive compounds from natural food sources (marine organisms, fish, milk, egg, algae, plant sources) due to their increased applications as safe alternatives to synthetic anti-oxidants in food and pharmaceutical industries.

Numerous peptides and polysaccharides with anti-oxidant activities have been identified and isolated from marine organisms for application as functional foods/health foods [24,25,26]. Peptides play an important role in the inhibition of free radicals and oxidative stress. It has been reported that the anti-oxidant mechanism of food originated peptides depends on composition, sequence and length [27,28]. Additionally, it was demonstrated that peptides with more hydrophobic (Ala, Val, Gly, Leu, Ile, Phe, Pro) amino acid residues can enhance anti-oxidant ability [15,29,30]. Most anti-oxidant peptides possess 3–15 amino acid residues [27,31,32,33,34]. Table 1 shows the molecular mechanisms of anti-oxidant peptides produced from various peanut worms.

There have been several investigations that reported the anti-oxidant capacity of peptides derived from peanut worms. The anti-oxidant activities exhibited by the peanut worms derived peptides were due to the inhibition of free radicals and enhancing the secretion of endogenous antioxidant enzymes such as SOD and glutathione peroxidase (GSH-Px). Zhu et al. [35] produced peptides from *Sipunculus nudus* by hydrolyzing with papain and found that peptides with molecular weight of 5868 Da showed excellent hydroxyl radical scavenging activity with 95% inhibition. Peptides generated from collagen of *Phascolosoma esculenta* have been reported to possess anti-oxidant capacity in vitro [36]. Liu et al. [15] prepared peptides (<3 kDa) from *Phascolosoma esculenta* by using pancreatin and investigated the anti-oxidant capacity of the peptides using mice model. It was found that peptide’s administration at 50, 100 and 150 mg/kg for 15 days dose-dependently improved the oxidative stress parameters such as GSH-Px, SOD, TAC and MDA in mice.

Apart from peptides, many studies have demonstrated that polysaccharides derived from peanut worms exhibit antioxidant activity through several ways including inhibition of DPPH, hydroxyl, superoxide free radicals, having reducing power, enhancing the antioxidant enzymes such as superoxide dismutase, glutathione peroxidase and upregulation of Nrf2 signaling pathway [16,19,37,38]. Anti-oxidant properties of polysaccharides produced from various peanut worms are presented in Table 2.

Researchers used various in vitro, and animal models to study the antioxidant capacity of polysaccharides derived from peanut worms. Studies conducted by Li et al. [39] and Qin et al. [37] documented that polysaccharides extracted from *Sipunculus nudus* showed a dose dependent inhibition of hydroxyl and superoxide radicals. Zhihao et al. [16] found that oligosaccharides from *Phascolosoma esculenta* exhibited anti-oxidative effects in sepsis mice model through the enhancement of enzyme activities of GSH-Px and SOD by activation of Nrf2 signaling pathway. Polysaccharide, composed of mannose, ribose, rhamnose, glucuronic acid, glucose, galactose, xylose, arabinose and fucose, derived from *Phascolosoma esculenta* have shown antioxidant activities in mice by enhancing superoxide dismutase (10–26%), glutathione peroxidase (11–30%) activities in serum and liver [38]. In a recent study, Yiqiao et al. [19] determined the radical scavenging activity and reducing power of polysaccharide isolated from *Phascolosoma esculenta* and reported the IC_50_ values of 0.567 and 0.605, 2.976 mg/mL respectively for DPPH and hydroxyl radicals and reducing power.

Most studies used in vitro and mice models for the determination of anti-oxidant activity of peptides and polysaccharides from peanut worms. However, cell culture and human studies are scanty. Therefore, further research is needed in humans and cells to verify the anti-oxidant benefits of these bioactive compounds of peanut worms.

## 3. Anti-Inflammatory Activities of Peptides and Polysaccharides Derived from Peanut Worms

Inflammation is a natural and complex defense mechanism of the immune system against a variety of harmful stimuli including pathogens, damaged cells, toxic chemicals, and irradiation. Generally, acute inflammation is beneficial to the body. However, chronic and uncontrolled inflammation can lead to various diseases such as cancer, diabetes, atherosclerosis, chronic kidney disease, arthritis and neurodegenerative disorders [40,41]. NSAIDs (non-steroidal anti-inflammatory drugs) are commonly used drugs for the treatment of inflammation. However, side effects associated with cardiovascular/renal/gastrointestinal systems have limited the application of NSAIDs. Therefore, recently there is a great interest in the use of food derived bioactive components (e.g., peptides and polysaccharides) for treatment or management of inflammation/inflammatory diseases due to their food origin and no/less side effects. Research has revealed that food derived bioactive compounds play an important role in the mitigation of inflammation/inflammatory diseases.

Numerous recent studies have shown that bioactive compounds (peptides, polyphenols, proteins, lipids and polysaccharides) derived from several food sources (fish, milk, egg, marine organisms and plant foods) possess anti-inflammatory properties [42,43,44,45,46]. Marine peanut worms have been investigated for anti-inflammatory compounds. Peptides and polysaccharides isolated from peanut worms have demonstrated to modulate the inflammatory response by inhibiting the production of pro-inflammatory mediators, TNF-α, IL-1β and TGF-β1, and reducing the activity of enzymes, cyclooxygenase-2 (COX-2) and inducible nitric oxide synthase (iNOS).

Recently, anti-inflammatory peptides have been identified and isolated from few peanut worms after hydrolysis with proteolytic enzymes. Peptides isolated from *Sipunculus nudus* have shown anti-inflammatory effects through the modulation of several mechanisms such as reduction of expression of pro-inflammatory mediators, TNF-α, IL-1β, IL-6, iNOS and COX-2 and inhibition of NO production [1,20]. The anti-inflammatory effects of peptides isolated from peanut worms are presented in Table 3.

The food originated anti-inflammatory peptides are usually 2–10 amino acids in length. In addition to the peptide’s length, composition and sequence of peptides also play an important role in anti-inflammatory activity [47,48]. In recent years, researchers have documented the extraction of anti-inflammatory peptides from peanut worm, *Sipunculus nudus.* Sangtanoo et al. [1] isolated two peptides, LSPLLAAH (821.48 Da) and TVNLAYY (843.42 Da), from *Sipunculus nudus* after hydrolysis with neutrase, flavourzyme, and Alcalase and reported that both peptides showed strong anti-inflammatory activity in LPS-stimulated RAW264.7 macrophages by decreasing the expression of pro-inflammatory mediators, iNOS, IL-6, TNF-α and COX-2 after treatment with 30, 60, 120 mM for 12 h. In another recent study, Lin et al. [20] produced collagen peptides from *Sipunculus nudus* by using animal hydrolytic protease and flavor protease and demonstrated that peptides inhibited inflammation in the wound of mice skin through the reduction of mRNA levels of TGF-β1, TNF-α and IL-1β.

Apart from the peptides, the anti-inflammatory effects of polysaccharides isolated from peanut worms have also been studied considerably in recent years (Table 4). Chen-Xiao et al. [2] investigated the anti-inflammatory activities of water soluble polysaccharides of *Sipunculus nudus* using different mouse and rat models of inflammation. It was found that polysaccharide treatment at 50, 100 and 200 mg/kgBW for 6 days dose-dependently reduced the inflammation of carrageenan-induced paw oedema, dextran-induced rat paw oedema, carrageenan-induced peritonitis, xylene-induced ear oedema, and acetic acid-induced vascular permeability in mice. Zhihao et al. [16] extracted oligosaccharides, composed of D-glucosyl, and D-galactosyl residues with α- and β-type linkages, from *Phascolosoma esculenta* and demonstrated that administration of oligosaccharides at 1, 10 and 5 mg/mL for 30 days noticeably decreased the production of IL-1β and TNF-α and enhanced IL-10 in mice with sepsis induced inflammation.

The anti-inflammatory properties shown by the peptides and polysaccharides of peanut worms could be due to the suppression of nuclear factor kappa B (NF-κB) activation. Inhibition of NF-κB activation decreases the expression of COX-2 and iNOS and production of TNF-α and IL-1β, and therefore reduces inflammation. Though the anti-inflammatory activities have been reported for the peptides and polysaccharides of peanut worms, the underlying molecular mechanisms of anti-inflammatory activity have not been explored extensively so far. Therefore, further research is needed to determine the exact molecular mechanisms of action of these anti-inflammatory bioactive components (peptides and polysaccharides) isolated from peanut worms. This would enhance the applications of these bioactive compounds in food and pharmaceutical industries.

## 4. Anti-Hypertensive Activity of Peptides from Peanut Worms

Hypertension (high blood pressure) is a serious medical condition that affects 1280 million people globally aged between 30–79 years. It has been reported that several diseases related to heart (myocardial infarction, coronary heart disease), brain (stroke) and kidney (kidney failure) are linked to hypertension. Angiotensin I-converting enzyme (ACE; EC 3.4.15.1) plays an important role in regulation of blood pressure. ACE cleaves the two C-terminal dipeptides of inactive angiotensin I to produce angiotensin II, which is a potent vasoconstrictor that prevents the catalytic function of bradykinin, a vasodilator. Recent research has shown that inhibition of ACE with food derived peptides could be an effective way in the prevention/management of hypertension. It has been demonstrated that numerous peptides isolated from various food sources (milk, egg, fish, meat, marine organisms and vegetables) have shown anti-hypertensive activity [49,50,51,52,53,54,55,56,57].

Recently, several researchers investigated the anti-hypertensive effects of peptides derived from peanut worms such as *Phascolosoma esculenta* and *Sipunculus nudus.* Anti-hypertensive activities of peptides originated from peanut worms have been widely studied due to their excellent bioactive properties and safety profiles. Pepsin and trypsin are the two proteolytic enzymes that are extensively used in the production of anti-hypertensive peptides from peanut worms [3,17,58,59,60,61]. These enzymes specifically cleave the large molecular weight proteins into smaller peptides. The large proteins are usually unable to bind the active site of ACE, however, the smaller peptides generated by enzymatic hydrolysis could easily bind to the active site of ACE as inhibitor and thereby prevent high blood pressure [3]. It has been demonstrated that peptides derived from peanut worms showed anti-hypertensive activity through several mechanisms including changing the secondary structure of ACE, competitive and non-competitive inhibition of ACE, reducing systolic blood pressure and binding to the active sites of ACE [17,58,59,60] (Table 5).

The ACE inhibitory/antihypertensive activity of food derived peptides depends on the composition, sequence and length of the peptides. Peptides with more hydrophobic amino acids (aromatic and branched chain aliphatic) have been demonstrated to inhibit ACE efficiently [3]. Most of the ACE inhibitory peptides isolated from peanut worms contain 2–11 amino acids residues [17,58,59,60,62]. Du et al. [60] used pepsin to cleave the water soluble protein of *Phascolosoma esculenta* in order to obtain low molecular weight peptides with potent angiotensin I-converting enzyme (ACE) inhibitory activity. A novel ACE inhibitory peptide, Ala-Trp-Leu-His-Pro-Gly-Ala-Pro-Lys-Val-Phe, was isolated with IC_50_ value of 135 M. The authors investigated the inhibitory kinetics of the peptide and found that the identified peptide inhibited ACE through competitive inhibition. Furthermore, the peptide (Ala-Trp-Leu-His-Pro-Gly-Ala-Pro-Lys-Val-Phe) at 10 mg/kg dose showed anti-hypertensive effects in spontaneously hypertensive rats (SHR) by significantly decreasing the systolic blood pressure (SBP) around 30 mmHg. Water-soluble and insoluble proteins of *P. esculenta* were extracted, by Wu et al. [3], and the fractions hydrolyzed with pepsin and trypsin. The hydrolysates exhibited ACE inhibitory activity with IC_50_ values between 0.1 to 0.67 mg/mL. It was demonstrated that the peptides derived from water-soluble and insoluble proteins significantly reduced both diastolic blood pressure (DBP) (23–45 mmHg) and systolic blood pressure (SBP) (20–33 mmHg) in spontaneously hypertensive rats after single oral administration at a dose of 1 g/kgBW. Protein from *Sipunculus nudus* was hydrolysed with Protamex and three ACE inhibitory peptides IND, VEPG, and LADEF were isolated with IC_50_ values of 34.72, 20.55 and 22.77 μmol/L, respectively [62]. In another investigation, Guo et al. [59] identified and isolated three anti-hypertensive peptides, RYDF, YASGR and GNGSGYVSR, from *Phascolosoma esculenta.* It was found that the three peptides inhibited ACE through non-competitive inhibition with IC_50_ values between 29–235 μM. Furthermore, GNGSGYVSR reduced systolic blood pressure by 31 mmHg at 2 h after oral administration in spontaneously hypertensive rats. Recently, two novel ACE inhibitory peptides, GNGSGYV and SR, were reported from *Phascolosoma esculenta* after hydrolysis with pepsin and trypsin. The two peptides showed synergistic effect on ACE inhibition with IC_50_ value of 170 μM. The synergistic mechanism indicated that SR and GNGSGYV significantly changed the secondary structure of ACE. It was also found that the peptide SR initially formed a coordinate bond with the catalytic Zn of ACE and then GNGSGYV attached with arginine of dipeptide (SR) and the amino acids of ACE active site through hydrogen bonds and thereby prevent the substrate attachment with ACE [17]. In addition to the traditional hydrolysis, virtual hydrolysis of peanut worms has been performed by several researchers in order to identify the potential ACE inhibitory peptides. Hongxi et al. [61] virtually hydrolyzed *Phascolosoma esculenta* protein with pepsin and trypsin and identified five ACE inhibitory peptides, AYF, EL, GLR, HK and ILK, with IC_50_ values in the range of 3.43–4.18 U/mL. In another study, Liu et al. [58] conducted virtual hydrolysis of *Phascolosoma esculenta* with pepsin, trypsin and a mixture of pepsin and trypsin for identification of ACE inhibitory peptides and predicted that 284 di- and tri-peptides from *Phascolosoma esculenta* possess ACE inhibitory activity with IC_50_ less than 50 μM.

The research indicates that anti-hypertensive peptides obtained from peanut worms could be used as a natural component for application in health foods/nutraceuticals to prevent/treat hypertension. However, further research is needed on the exact molecular mechanisms of action of these anti-hypertensive peptides.

## 5. Immunomodulatory Activity of Polysaccharides Derived from Peanut Worms

Immune system is a complex defense network that protects the body against invading pathogens such as bacteria, viruses, fungi, protozoans, and prevents the growth of cancer cells. Immunomodulators are compounds that can potentially suppress or stimulate the immune system of host by modulating various immune cells and/or their signaling molecules (e.g., T cells, B cells, macrophages, NK cells, dendritic cells and cytokines) [63]. Recently, several polysaccharides with immunomodulatory activity have been identified from various food sources including mushrooms, fruits, cereals and algae [13].

Recent research has indicated that polysaccharides stimulate various immune cells (e.g., macrophages, NK cells, dendritic cells) by binding to the cell surface receptors such as Toll-like receptor 4 (TLR4), cluster of differentiation 14 (CD14), scavenger receptor (SR), complement receptor 3 (CR3), mannose receptor (MR) and Dectin-1 [13,63].

It has been demonstrated that polysaccharides derived from peanut worms exert immunomodulatory effects through various mechanisms such as activation of macrophages, increasing the indexes of immune organs (thymus and spleen), enhancing the secretion of cytokines, and improving the phagocytosis function of macrophages and NK cell activity [18,64,65,66]. Table 6 shows the immunomodulatory activities of polysaccharides derived from peanut worms.

A number of studies reported the immune stimulating activity of polysaccharides extracted from peanut worms, *Sipunculus nudus* and *Phascolosoma esculenta*. Liang et al. [64] investigated the immunomodulatory effects of polysaccharides from *P. esulenta* using a mice model and found that administration of polysaccharides at 3.0, 6.0, 9.0 mg/kgBW for 2 months considerably stimulated the Con-A activated mouse spleenocytes and increased the index of liver, spleen and thymus of mice. A water soluble polysaccharide, composed of rhamnose (28%), fucose (16%) and galactose (56%), extracted from *Sipunculus nudus* exhibited immunomodulatory effects by activating macrophages through the upregulation of expression of cytokines, IL-6 and TNF-α, and inducing the expression of iNOS and COX-2 [65]. Li et al. [66] reported that polysaccharides from *Sipunculus nudus* enhanced the cellular and humoral immunity in mice by increasing the phagocytosis function and NK cell activity. Su et al. [67] used hepatoma (HepG2) bearing mice model to study the immunomodulatory effects of polysaccharides derived from *Sipunculus nudus*. The mice were treated with 50, 100, and 200 mg/kg polysaccharide for 30 days and it was found that the polysaccharide composed of L-rhamnose, L-arabinose, D-ribose, D-glucose and D-galactose showed immune stimulating effects through increase of thymus and spleen indexes, and upregulated the IL-2, IFN-γ, and TNF-α cytokines in serum of mice. In another recent study, Su et al. [18] demonstrated that polysaccharide of *Sipunculus nudus* increased the index of immune organs and augmented the secretion of cytokines, IL-2, IFN-γ and TNF-α, in hepatoma mice after treatment with 50, 100 and 200 mg/kg polysaccharides for 16 days. These results suggest that immunomodulatory polysaccharides from peanut worms have great potential for application as nutraceutical/health foods.

Although immunomodulatory activities of polysaccharides derived from peanut worms have been documented, the molecular mechanisms of action and structure-function relationship of these polysaccharides have not been investigated. Thus, more research is needed on molecular mechanisms to decipher the immunomodulatory effects of these polysaccharides. Additionally, the immune stimulatory effects of peptides derived peanut worms need to be investigated considerably in order to enhance their application as health foods.

## 6. Anti-Cancer Activities of Polysaccharides Derived from Peanut Worms

Cancer is one of the major causes of death worldwide. Approximately 10 million deaths were occurred in 2020 due to various cancers [68]. Cancer is uncontrolled growth of cells in the body. The cancer cells attack the adjacent normal cells and spread to the other parts of the body. Currently, many different chemo drugs are used in the cancer treatment. However, side effects (neutropenia, nausea, vomiting, hair loss, blood clots etc.) associated with these drugs have limited their usage. Additionally, several chemotherapy drugs kill the normal cells along with the cancer cells. Therefore, recently there is great interest in finding new anti-cancer agents with less side effects from natural sources such as spices, vegetables, marine sources etc. [69,70]. Polysaccharides isolated from numerous natural resources including plant and marine sources have been reported to have inhibition activity on malignant cells primarily through the induction of apoptosis [71,72].

Polysaccharides extracted from peanut worms have been demonstrated to possess anti-cancer activity against cancer cells and in vivo animal models. Most researchers determined the anti-cancer effects of peanut worm derived polysaccharides in hepatocellular carcinoma. Scientific evidence from recent studies showed that polysaccharides derived from peanut worms exhibited anti-cancer effects by modulating numerous molecular mechanisms including preventing the DNA synthesis, increasing the expression of pro-apoptosis proteins, TNF-α, caspase-3, and Bax, decreasing the expression of the anti-apoptosis proteins, survivin, Bcl-2, and VEGF, up-regulation of caspase-3, caspase-8, and caspase-9, enhancing the expression of ATF4, DDIT3, and IkBα and reduction of CYR61, HSP90, and VEGF expression [18,67,73]. The mechanisms of action of anticancer activities of polysaccharides derived from peanut worms are presented in Table 7.

Polysaccharides isolated from peanut worm *Sipunculus nudus* have shown anti-cancer effects in cultured cancer cells and mice models with transplanted cancer cells. Jie et al. [73,74] studied the molecular mechanisms of anticancer effects of polysaccharides, extracted from *Sipunculus nudus*, after treating the Hepg2.2.15 cells with 0.13, 0.25, 0.5 and 1 mg/mL for 24 and 48 h and reported that *Sipunculus nudus* derived polysaccharides induced dose-dependent apoptosis on Hepg2.2.15 cells by increasing the expression of pro-apoptosis proteins, TNF-α, caspase-3, and Bax, and decreasing the expression of the anti-apoptosis proteins, survivin, Bcl-2, and VEGF. Su et al. [67] investigated the anti-cancer effects of polysaccharides of *Sipunculus nudus* using HepG2 cells-bearing mice model and demonstrated that administration of polysaccharides at 50, 100 and 200 mg/kg for 1 month significantly inhibited the growth of HepG2 cells through increasing the expression of ATF4, DDIT3 and IkBα and down-regulation of CYR61, HSP90 and VEGF expression. In another recent study, Su et al. [18] reported that polysaccharides extracted from *Sipunculus nudus* showed anti-cancer effects in HepG2-bearing mice by inducing the apoptosis of tumor cells through the up-regulation of caspase-3, caspase-8, caspase-9 and Bax, and down-regulation of B-cell lymphoma-2 and vascular endothelial growth factor protein expression.

The above scientific evidence confirmed that polysaccharides of peanut worms could inhibit the growth of the cancer cells. However, there are various limitations that could hamper the use of these polysaccharides for human applications as nutraceuticals/drugs. Firstly, more reliable and consistent scientific evidence from clinical studies about the beneficial effects of these polysaccharides is needed before its use for the treatment/management of cancer. Secondly, data about cytotoxic effects on normal cells and in-depth knowledge of exact molecular mechanisms of anti-cancer activity of peanut worms derived polysaccharides are required from cell culture, animal and clinical studies. Additionally, most studies investigated the anticancer activity of polysaccharides isolated from *Sipunculus nudus*, although there are many peanut worm species in phylum, Sipuncula. Therefore, anti-cancer activity for polysaccharides of other peanut worm species needs to be explored.

## 7. Other Bioactivities of Peanut Worm Derived Peptides and Polysaccharides

Apart from above mentioned bioactive properties, many researchers documented various other bioactive properties including wound healing capacity, memory improvement and anti-hypoxia activity for peanut worm derived peptides and polysaccharides (Table 8 and Table 9). Chen-Xiao and Zi-Ru [14] used various hypoxia mice models to determine the anti-hypoxia activity of polysaccharides extracted from *Sipunculus nudus* and revealed that polysaccharides showed significant anti-hypoxic activity on normobaric hypoxia, chemical intoxicant hypoxia and acute cerebral ischemia hypoxia models after treatment with 10, 30, 100 mg/kg for 6 days. Liu et al. [15] produced peptides < 3 kDa from *Phascolosoma esculenta* by using pancreatin and found that administration of peptides at 50, 100 and 150 mg/kg for 15 days improved the spatial learning and memory ability in mice through the up-regulation of NR2A, NR2B, BDNF and CREB mRNA expressions in hippocampus. Lin et al. [20] investigated the wound healing properties of collagen peptides derived from *Sipunculus nudus* and demonstrated that the peptides clearly improved the healing rate and inhibited scar formation in mice by enhancing collagen deposition and inhibition of TGF-β/Smads signaling pathway.

## 8. Conclusions

In this review, the various bioactive properties of peptides and polysaccharides originated from peanut worms were summarized. Peanut worms derived bioactive compounds (peptides and polysaccharides) exhibited anti-oxidant, anti-inflammatory, immunomodulatory, anti-hypertensive, anti-cancer and wound healing activities through the modulation of various molecular mechanisms. Most researchers investigated these bioactivities using in vitro, cell culture and animal models. Clinical studies confirming these bioactivities of peptides and polysaccharides of peanut worms are scanty in literature. Therefore, more clinical investigations are needed to enhance the applications of these bioactive compounds of peanut worms. Additionally, in-depth molecular mechanisms of action, bioavailability and safety profiles of these bioactive compounds of peanut worms need to be investigated thoroughly. Moreover, peptides produced from peanut worms could be further explored for their potential anti-diabetic, anti-microbial, anti-obesity and anti-atherosclerosis activities. Although there are several species in phylum sipuncula, only two species (*Sipunculus nudus* and *Phascolosoma esculenta*) have been investigated extensively with regard to the bioactive compound’s identification and isolation. Hence, other species of peanut worms could also be studied for possible identification of bioactive compounds for various human applications.

## Figures and Tables

**Table 1:** Molecular mechanisms of anti-oxidant peptides derived from peanut worms.

Peanut Worm Name	Enzyme Used to Produce Peptides	Peptide Sequence and Molecular Weight	In Vitro/Cell Culture/Animals/Humans Used for the Study	Dose and Duration	Mechanism of Action/ Activities/Effects Showed	Ref.
*Sipunculus nudus*	Papain	Peptides 5868 Da	In vitro hydroxyl Radical scavenging activity	-------	The polypeptide showed great hydroxyl radical scavenging activity with 95.42% inhibition.	[35]
*Phascolosoma esculenta*	Pancreatin	Peptides < 3 kDa	Mice	50, 100 and 150 mg/kg for 15 days	Peptides dose-dependently improved the oxidative stress status (GSH-Px, SOD, TAC andMDA) in mice	[15]
*Phascolosoma esculenta*	Papain	-----------	In vitro total anti-oxidant capacity	---------	Collagen peptides from *Phascolosoma esculenta* showed total anti-oxidant capacity with 3.8 U/mg	[36]

**Table 2:** Molecular mechanisms of anti-oxidant activities of polysaccharides derived from various peanut worms.

Source	Composition of Polysaccharide Extract	Cell Culture/ Animal Models	Dose and Duration	Molecular Mechanisms/Effects	Ref.
*Sipunculus nudus*	Polysaccharide was com-posed of mannose, rhamnose, galacturonic acid, glucose, arabinose and fucose	In vitro hydroxyl radical activity.	0.25, 0.5, 1.0, 2.0, 5.0,10.0, 20.0 mg/mL for 30 min	Polysaccharide showed powerful scavenging activity on hydroxyl radical in a dose dependent manner.	[39]
*Sipunculus nudus*	-----------------	In vitro reducing power, hydroxyl and superoxide radicals inhibition assay	200–1000 µg/mL for 30 min	*S. nudus* polysaccharides showed dose dependent inhibition of hydroxyl and superoxide radicals and exhibited great reducing power.	[37]
*Phascolosoma esculenta*	D-glucosyl, D-galactosyl, with small amount of D-mannosyl, D-arabinosyl and residues with a- and b-type linkage.	Mice	1, 10 and 5 mg/mL for 30 days	Oligosaccharides from *Phascolosoma esculenta* enhanced the enzyme activities of GSH-Px and SOD by upregulating Nrf2 mRNA expression in sepsis mice model.	[16]
*Phascolosoma esculenta*	Mannose, ribose, rhamnose, glucuronic acid, glucose, galactose, xylose, arabinose and fucose	In vitro DPPH, superoxide anion, hydroxyl radicals and ferrous ion chelating and mice model	0.2, 0.4 and 0.8 g/kgBW for 25 d	Polysaccharides from *Phascolosoma esculenta* scavenged free radicals dose-dependently and showed antioxidant activities in mice by enhancing superoxide dismutase (SOD) (10.2–22.2% and 18.8–26.9%), glutathione peroxidase (GSH-Px) (11.9–15.4% and 26.6–30.4%) activities in serum and liver.	[38]
*Sipunculus nudus*	-----------	In vitro DPPH and hydroxyl radical scavenging activities	0.2 mg/mL	Free radical scavenging rates increased significantly with the increase of concentration. The scavenging activities of hydroxyl radical and DPPH radical were found to be 12.58% at the concentration of 0.20 mg/mL.	[39]
*Phascolosoma esculenta*	polysaccharide contained glucose with acetylaminoand pyran rings and connected by *α*-glycosidic bonds	In vitro reducing power, DPPH and hydroxyl radical scavenging activities	1,5,10,15,20, and 25 mg/mL for 30 min	Polysaccharide showed DPPH and hydroxyl radical scavenging and reducing power with IC_50_ of 0.567 and 0.605, 2.976 mg/mL, respectively.	[19]

**Table 3:** Molecular mechanisms of anti-inflammatory peptides derived from peanut worms.

Peanut Worm Name	Enzyme Used to Produce Peptides	Peptide Sequence and Molecular Weight	In Vitro/Cell Culture/Animals/Humans Used for the Study	Dose and Duration	Mechanism of Action/Activities/Effects Showed	Ref.
*Sipunculus nudus*	Neutrase, Flavourzyme, and Alcalase	LSPLLAAH (821.48 Da) and TVNLAYY (843.42 Da).	RAW 264.7 macrophages	30, 60, 120 mm for 12 h	Peptides (LSPLLAAH and TVNLAYY) inhibited NO production and decreased the expression of pro-inflammatory mediators, iNOS, IL-6, TNF-α, and COX-2, in LPS-stimulated RAW264.7 macrophages.	[1]
*Sipunculus* *nudus*	Animal hydrolytic protease (3000 U/g) and flavor protease	Collagen peptides < 5 kDa	Mice	2 g/mL for 7 days	Peptides showed anti-inflammatory activity through the reduction of mRNA levels of TGF-β1, TNF-α and IL-1β in the wound of mice skin.	[20]

**Table 4:** Molecular mechanisms of anti-inflammatory activity of polysaccharides derived from various peanut worms.

Source	Composition of Polysaccharide Extract	Cell Culture/ Animal Models	Dose and Duration	Molecular Mechanisms/Effects	Ref.
*Sipunculus nudus*	Water extract	Mouse and rat oedema paw models	50, 100 and 200 mg/kg for 6 days	Water extract from the body wall of *Sipunculus nudus* showed dose-dependent anti-inflammatory activity in the carrageenan-induced paw oedema, dextran-induced rat paw oedema, cotton pellet granuloma, carrageenan-induced peritonitis, xylene-induced ear oedema, and acetic acid-induced vascular permeability models.	[2]
*Phascolosoma esculenta*	D-glucosyl, D-galactosyl, with small amount of D-mannosyl, D-arabinosyl and residues with α- and β-type linkage.	Mice	1, 10 and 5 mg/mL for 30 days	Oligosaccharides from *Phascolosoma esculenta* considerably decreased the secretion of IL-1β and TNF-α and enhanced the IL-10 in sepsis mice.	[16]

**Table 5:** Molecular mechanisms of action of ACE-inhibitory/anti-hypertensive peptides derived from various peanut worms.

Peanut Worm Name	Enzyme Employed to Produce Peptides	Peptide Sequence and Molecular Weight	In Vitro/Cell Culture/Animals/Humans Used for the Study	IC_50_/EC_50_ Values	Activity/Mechanisms of Action Showed	Ref.
*Phascolosoma esculenta*	Pepsin	AWLHPGAPKVF	In vitro ACE inhibition assay & spontaneously hypertensive rats	IC_50_ value of 135 M	Peptide inhibited ACE through competitive inhibition and exhibited anti-hypertensive effects in rats by significantly reducing the systolic blood pressure around 30 mmHg.	[60]
*Phascolosoma esculenta*	Pepsin and trypsin	----------------	In vitro ACE inhibition assay & spontaneously hypertensive rats	IC_50_ values of 0.67 and 0.24 mg/mL	Peptides significantly reduced both diastolic blood pressure (DBP) and systolic blood pressure (SBP) and inhibited ACE in vitro.	[3]
*Phascolosoma esculenta*	Pepsin, and trypsin	AYF, EL, GLR, HK, and ILK	In vitro ACE inhibition assay	IC_50_ values of 3.43–4.18 U/ml	Peptides exhibited ACE inhibitory activity with IC_50_ values in the range of 3.43–4.18 U/mL	[61]
*Phascolosoma esculenta*	Pepsin and trypsin	284 di- and tri-peptides	In vitro ACE inhibition assay	IC_50_ less than 50 μM	Peptides inhibited the ACE.	[58]
*Sipunculus nudus*	Protamex	IND, VEPG, LADEF	In vitro ACE inhibition assay	IC_50_ values for ACE inhibition were 34.72, 20.55 and 22.77 μmol/L	The peptides IND, VEPG, and LADEF showed ACE inhibition activity with IC_50_ values of 34.72, 20.55 and 22.77 μmol/L, respectively.	[62]
*Phascolosoma esculenta*	Pepsin and trypsin	RYDF, YASGR and GNGSGYVSR	In vitro ACE inhibition assay & spontaneously hypertensive rats	IC_50_ values of 235, 184 and 29 μM respectively for RYDF, YASGR and GNGSGYVSR	Three peptides inhibited ACE through non-competitive inhibition. GNGSGYVSR reduced systolic blood pressure 31 mmHg at 2 h after oral administration in spontaneously hypertensive rats.	[59]
*Phascolosoma esculenta*	Pepsin and trypsin	GNGSGYV and SR	In vitro ACE inhibition assay	IC_50_ value of 170 μM	GNGSGYV and SR showed ACE inhibition through synergistic effect. SR initially attacked the catalytic Zn of ACE and formed coordinate bond, and then GNGSGYV attached with the residues of ACE active site by hydrogen bonds.	[17]

**Table 6:** Molecular mechanisms of immunomodulatory activity of polysaccharides derived from peanut worms.

Source	Composition of Polysaccharide Extract	Cell Culture/ Animal Models	Dose and Duration	Molecular Mechanisms/Effects	Ref.
*Phascolosoma esculenta*	------------	Mice model	3.0, 6.0, 9.0 mg/kgBW for 2 months	Polysaccharides from *P. esulenta* significantly enhanced liver, spleen and thymus index of mice and increased Con A-stimulated mouse spleen cells.	[64]
*Sipunculus nudus*	Monosaccharide composition -rhamnose (28%), fucose (16%) and galactose (56%)	Murine macrophages from BALB/c mice and human macrophages	5–80 µg/mL for 24 h	The water soluble polysaccharide isolated from *S. nudus* showed immunostimulating activity by activating macrophages through the upregulation of expression of cytokines, IL-6 and TNF-α, and inducing the expression of iNOS and COX-2.	[65]
*Sipunculus nudus*	------------	Mice	------------	Polysaccharides from *Sipunculus nudus* promoted the cellular immunity and humoral immunity through the enhancing the phagocytosis function and NK cell activity in mice.	[66]
*Sipunculus nudus*	L-rhamnose, Larabinose, D-ribose, D-glucose and D-galactose	Hepatoma HepG2-bearing Mice	50,100, and 200 mg/kg, 1 month	Polysaccharide extract from *Sipunculus nudus* enhanced the immune response through increase of thymus and spleen indexes, and upregulating the IL-2, IFN-γ, and TNF-α cytokines in serum of mice.	[67]
*Sipunculus nudus*	Repeating units are →3,4-β-D-GlcpNAC (1→ and →4)-α-D-Glcp (1→ in the ratio of 15:1; →2)-α-D-Galp-(1→ as a side chain; and β-D-Galp-(1→ and α-D-Glcp-(1→ as end groups	hepatoma HepG2-bearingmice	50,100, and 200 mg/kg, 16 days	Polysaccharide increased the index of immune organs and augmented the secretion of cytokines IL-2, IFN-γ and TNF-α.	[18]

**Table 7:** Molecular mechanisms of anti-cancer activity of polysaccharides derived from various peanut worms.

Source	Composition of Polysaccharide Extract	Cell Culture/ AnimalModels	Dose and Duration	Molecular Mechanisms/Effects	Ref.
*Sipunculus nudus*	Extractcontains 35.3% neutral sugar, including Ara 10.7%, Rha 12.6%, Gal 16.4%, Glu 31.3%, Xyl 18.2%, and Man 10.8%.	Hepg2.2.15 cells	0.13, 0.25, 0.5, and 1 mg/mL for 24 and 48 h	Polysaccharides showed anti-cancer activities by preventing the DNA synthesis of Hepg2.2.15 cells and increasing the expression of pro-apoptosis proteins, TNF-α, caspase-3, and Bax, and decreasing the expression of the anti-apoptosis proteins survivin, Bcl-2, and VEGF.	[73]
*Sipunculus nudus*	L-rhamnose, Larabinose, D-ribose, D-glucose and D-galactose	Hepatoma HepG2-bearing Mice	50,100, and 200 mg/kg, 1 month	Polysaccharides showed anti-tumor activity by inhibiting the growth of HepG2 cells through increase of ATF4, DDIT3, and IkBα expression and decrease of CYR61, HSP90, and VEGF expression.	[67]
*Sipunculus nudus*	Repeating units of →3,4-β-D-GlcpNAC (1→ and →4)-α-D-Glcp(1→ in the ratio of 15:1; →2)-α-D-Galp-(1→ as a side chain; and β-D-Galp-(1→ and α-D-Glcp-(1→ as end groups	hepatoma HepG2-bearing mice	50,100, and 200 mg/kg, 16 days	Extracted polysaccharide enhanced the apoptosis of tumour cells through the mitochondrial apoptosis pathway by upregulating caspase-3, caspase-8, caspase-9 and BCL2-associated X, and downregulating B-cell lymphoma-2 and vascular endothelial growth factor protein expression.	[18]

**Table 8:** Anti-hypoxia activity of polysaccharides derived from various peanut worms.

Source	Composition of Polysaccharide Extract	Cell Culture/ Animal Models	Dose and Duration	Molecular Mechanisms/Effects	Ref.
*Sipunculus nudus*	Rhamnose (28%), fucose (16%) and galactose (56%).	Mice model	10, 30, 100 mg/kg for 6 days	The extracted polysaccharide exhibited significant anti-hypoxic activity on normobarie hypoxia, chemical intoxicant hypoxia and acute cerebral ischemia hypoxia models in mice.	[14]

**Table 9:** Effects of peptides derived from peanut worms on wound healing and spatial learning and memory.

Peanut Worm Name	Enzyme Used to Produce Peptides	Peptide Sequence and Molecular Weight	In Vitro/Cell Culture/Animals/Humans Used for the Study	Dose and Duration	Mechanism of Action/Activities/Effects Showed	Ref.
*Sipunculus* *nudus*	Animal hydrolytic protease (3000 U/g) and flavor protease (3000 U/g)	Collagen peptides < 5 kDa	human umbilical vein endothelial cells (HUVEC), human immortalized keratinocytes (HaCaT) and human skin fibroblasts (HSF) and mice	2 g/mL for 28 days and 500 µg/mL for 12, 24, 30, 36 h	Collagen peptides derived from *Sipunculus nudus* exhibited great capacity to induce HUVEC, HaCaT and HSF cells proliferation and migration in vitro. Peptides noticeably improved the healing rate and inhibited scar formation in mice through the mechanisms of reducing inflammation, enhancing collagen deposition and recombination and blockade of the TGF-β/Smads signal pathway.	[20]
*Phascolosoma esculenta*	Pancreatin	Peptides < 3 kDa	Mice	50, 100 and 150 mg/kg for 15 days	Peptides improved the spatial learning and memory ability doses-dependently through the up-regulation of NR2A, NR2B, BDNF and CREB mRNA expressions in hippocampus of mice. 100 mg/kg group showed better performance in spatial learning and memory compared with 50, and 150 mg/kg.	[15]

## Data Availability

Not applicable.

## Abbreviations

ACE: Angiotensin I-converting enzyme; ATF4: Activating transcription factor 4; Bcl-2: B-cell lymphoma 2; BDNF: Brain Derived Neurotrophic Factor; CD14: cluster of differentiation 14; COX-2: cyclooxygenase-2; CR3: complement receptor 3; CREB: cAMP responsive element binding protein; CYR61: Cysteine-rich angiogenic inducer 61; DBP: diastolic blood pressure; DDIT3:DNA Damage Inducible Transcript 3; DNA: Deoxyribonucleic acid; DPPH: 2,2-diphenyl-1-picrylhydrazyl; GPx: Glutathione peroxidase; GSH-Px: glutathione peroxidase; HaCaT: human immortalized keratinocytes; HSF: human skin fibroblasts; HSP90: Heat shock protein 90; HUVEC: human umbilical vein endothelial cells; IFN-γ: Interferon gamma; IL: interleukin; iNOS: inducible nitric oxide synthase; MDA: malondialdehyde; mRNA: messenger ribonucleic acid; MR: mannose receptor; NF-κB: nuclear factor kappa B; NK cells: Natural killer cells; NO: Nitric oxide; Nrf2: nuclear factor erythroid 2-related factor; NSAIDs: non-steroidal anti-inflammatory drugs; ROS: reactive oxygen species; SBP: systolic blood pressure; SR: scavenger receptor; SOD: superoxide dismutase; TAC: Totalantioxidantcapacity; TGF-β1: Transforming growth factor beta1; TLR4: Toll-like receptor 4; TNF-α: Tumour Necrosis Factor alpha; VEGF: Vascular endothelial growth factor.
